# Should Finasteride Be Routinely Given Preoperatively for TURP?

**DOI:** 10.1155/2013/458353

**Published:** 2013-07-29

**Authors:** O. M. Aboumarzouk, M. Z. Aslam, A. Wedderburn, K. Turner, O. Hughes, H. G. Kynaston

**Affiliations:** ^1^Department of Urology, University Hospital of Wales, Cardiff CF14 4XW, UK; ^2^Royal Bournemouth Hospital, Bournemouth, UK

## Abstract

*Objective*. The aim of the review was to compare the use of finasteride to placebo in patients undergoing TURP procedures. *Material & Methods*. We searched the Cochrane Central Register of Controlled Trials (CENTRAL), MEDLINE (1966–November 2011), EMBASE (1980–November 2011), CINAHL, Clinicaltrials.gov, Google Scholar, reference lists of articles, and abstracts from conference proceedings without language restriction for studies comparing finasteride to placebo patients needing TURPs. *Results*. Four randomised controlled trials were included comparing finasteride to a placebo. A meta-analysis was not conducted due to the disparity present in the results between the studies. Three of the studies found that finasteride could reduce either intra- or postoperative bleeding after TURP. One study found finasteride to significantly lower the microvessel density (MVD) and vascular endothelial growth factor (VEGF). None of the studies reported any long-term complications related to either the medication or the procedure. *Conclusion*. finasteride reduces bleeding either during or after TURP.

## 1. Introduction

Bladder outflow obstruction (BOO) due to benign prostatic hyperplasia (BPH) is the commonest urological condition affecting men over 50 years of age. Medical therapy is usually the first line management of BPH. finasteride, a 5-alpha reductase inhibitor (5ARI), blocks the conversion of testosterone into the more potent dihydrotestosterone (DHT) and has been shown to reduce blood levels by 80–85% in 1-2 weeks [1–3]. By the suppression of DHT, finasteride reduces prostatic tissue growth by decreased glandular and fibromuscular tissue and has been shown to reduce the overall size of the prostate by 30% within 6–12 months [2, 4]. Furthermore, studies have found that 5ARI also suppresses the androgen controlled vascular endothelial growth factor (VEGF), leading to decreased angiogenesis and less prostatic bleeding [2, 5, 6]. 

Transurethral resection of the prostate (TURP) is the gold standard modality of treatment for BPH where medical therapy has failed or when there is a risk to renal function due to BOO. Though, TURP is an established procedure, significant intraoperative as well as postoperative bleeding remains a common complication leading to postoperative clot retention and blood transfusion [7–11]. In a prospective study, Hagerty et al. were first to report a reduction in blood loss during TURP in patients taking finasteride preoperatively [8]. Since then numerous randomised controlled trials (RCTs) have also emerged to report similar findings [1, 7, 10, 11]. Despite this, in a UK-based survey of British urologists, though 98% said they commonly use finasteride for haematuria thought to be prostatic in origin, only 4% use it before TURP [12]. Furthermore, the American Urological Association's (AUA) guidelines state that there is insufficient evidence to recommend perioperative 5ARI treatment to decrease bleeding [13].

Therefore, we aimed to conduct a Cochrane level systematic review of the literature to establish the role of finasteride use prior to TURP.

## 2. Methodology

### 2.1. Search Strategy and Study Selection

The systematic review was performed according to the Cochrane reviews guidelines. The search strategy was conducted to find relevant studies from MEDLINE (1990–August 2011), EMBASE (1990–August 2011), Cochrane Central Register of Controlled Trials (CENTRAL) (in The Cochrane Library-Issue 1, 2011), CINAHL (1990–August 2011), Clinicaltrials.gov, Google Scholar, and individual urological journals.

Terms used included “5-alpha reductase inhibitor,” “TURP,” “Transurethral resection of the prostate,” “5ARI,” and “finasteride.”

Mesh phrases included

- ((“Randomized Controlled Trial” [Publication Type]) AND “Transurethral Resection of Prostate” [Mesh]) AND “finasteride” [Mesh],
- (“Transurethral Resection of Prostate” [Mesh]) AND “finasteride” [Mesh],
- (“finasteride” [Mesh]) AND “Randomized Controlled Trial” [Publication Type],
- (“Randomized Controlled Trial” [Publication Type]) AND “Transurethral Resection of Prostate” [Mesh].

Papers in languages other than English were included if data was extractable, and also references of searched papers were evaluated for potential inclusion. Authors of the included studies were contacted wherever the data was not available or not clear.

Two reviewers identified all studies that appeared to fit the inclusion criteria for full review. Each reviewer independently selected studies for inclusion in the review. Disagreement between the two extracting authors was resolved by consensus. 

### 2.2. Data Extraction and Analysis

Studies reporting on finasteride use prior to TURP procedures were included. The main outcome parameters were estimated blood loss and decrease in haemoglobin (Hb) (difference between the pre- and postoperative Hb). Secondary outcomes were age, PSA, resection weight, blood loss per gram of resected tissue, pathology result of prostate cancer, microvessel density (MVD), and vascular endothelial growth factor (VEGF).

Main exclusion criteria were men previously on 5ARI, history of prostate cancer, with a history of bleeding diathesis, or on active anticoagulant therapy.

We could not pool the data into a meta-analysis as there were no comparable parameters or outcome measures in any of the studies. We attempted to contact the corresponding authors of all included studies; however no reply was received. Therefore, we have done a descriptive analysis of the studies.

### 2.3. Quality Assessment

An assessment of the methodological quality of the included studies into the meta-analysis was conducted in line with the Cochrane handbook [14]. For quality assessment the selection bias, performance bias, detection bias, attrition bias, and reporting bias were assessed in each of the included studies. We used Review Manager (RevMan 5.0.23) to plot the quality assessment.

## 3. Results

**Figure 1:**
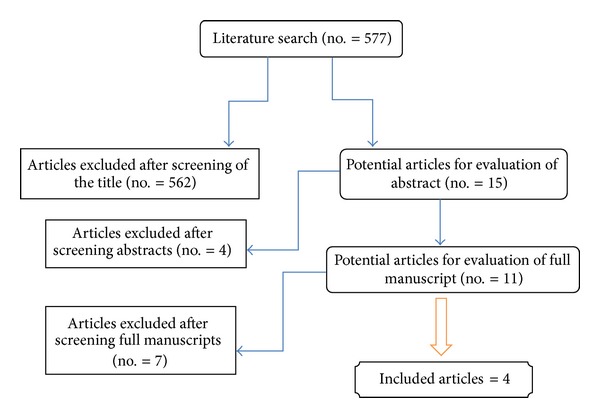
Flowchart for article selection process of the review.

The search strategy yielded 577 hits, with 562 articles excluded from the title and 4 from the abstract, due to nonrelevance to the aim of this review (Figure 1). Of the remaining 11 studies, 4 were included [1, 7, 10, 11] and 7 excluded [2–6, 8, 12]. Six of the seven excluded studies did not examine the role of finasteride to reduce blood loss during TURP procedures [2–6, 12]. While Hagerty et al. was the first to report on the subject, the study was a prospective report and not a randomised study [8]. 

### 3.1. Characteristics of the Included Studies

All the studies examine the role of finasteride to reduce blood loss during TURP procedures [1, 7, 10, 11]. A total of 109 patients in the finasteride group and 118 patients in the placebo group were included. All studies were published between 2001–2005. Two of the studies, by the same urology firm, compared 5 mg of finasteride to a placebo given 2 weeks prior to the TURP [1, 7], while one study gave finasteride 4 weeks prior [10], and the last study gave it 12 weeks prior TURP [11].

Table 1 depicts the main results of each of the studies. One of the studies looked at the effect of finasteride on VEGF and MVD [1], while another looked at MVD alone [11]. Three studies reported on the total blood loss as well as the blood loss per resection weight [7, 10, 11]. In addition to the patient's demographics and the blood loss, all studies compared the resection weight of the prostate chippings and PSA levels between the two groups. Three studies reported their histologically confirmed prostate cancer [1, 7, 11]. One study reported if the patients were catheterised prior to TURP [1]. All four studies reported their complication rates.

Though most of the studies reported on the same outcome parameters, each study reported the results differently (Table 1). Sandfeldt et al. reported their findings in median (range) [11], Donohue et al. reported using mean (range) in one of their papers and mean (% CI) in the other [1, 7], while Ozdal et al. paper's one was the only to report their findings as means ± standard deviation. None of the authors responded to an attempt to gather their data for a meta-analysis. 

### 3.2. Quality Assessment

The overall assessment showed a low risk of bias by most studies. Though all four studies were randomised, none of them mentioned how the randomisation sequence was generated or how the allocation concealment was done (Table 2). Three studies were blinded while Ozdal et al. did not mention whether or not there was blinding in their study. No other risk of bias was identified. However, a confounding issue does rise with regards to one study [1]. The authors have previously published results of an RCT that looked at the role of finasteride and bleeding during TURP, and whether or not the same cohort of patients were used for the second paper was not clear [1, 7]. Despite this, their second paper looked only at MVD and VEGF, with one extra patient in the finasteride group and two in the placebo group (Table 1) [1]. With no clarification from the corresponding author and an unclear timing of the conducted study, this issue was deemed to have a high risk of bias (Table 2).

### 3.3. Effects of Intervention

Though none of the studies reported any significant difference with the resected prostate weight, two of the three studies reported significantly less blood loss/resection tissue in the finasteride group [7, 10], while the third study, in a subgroup analysis, found that with finasteride treatment larger sized prostates did bleed significantly less [11]. The fourth study found that there was a significant reduction in both MVD and VEGF in patients pretreated with finasteride [1]. 

Ozdal et al. found that the finasteride group had less bleeding and lower Hb. While Donohue et al. found no difference in bleeding intraoperatively, there was significantly more Hb loss in the placebo group compared to the finasteride groups postoperatively. Similarly, Sandfeldt et al. found significantly less blood loss in the finasteride group in larger (≥18 g) prostates.

No long-term side effect (>3 months) was experienced in any of the studies. In total 4/118 patients required a blood transfusion in the placebo group compared to none in the finasteride group. 

## 4. Discussion

Although a meta-analysis was not feasible, the evidence from the four RCTs portrays some benefit in use of finasteride preoperatively (Table 1). Three of the studies have shown that finasteride can reduce either intra- or postoperative bleeding after TURP [7, 10, 11]. None of the studies reported any long lasting (>3 months) complications related to either the medication or the procedure.

Finasteride has been proven to reduce 77–100% of the amount of haematuria related to prostatic bleeding when taken daily [15]. Numerous studies have shown that finasteride, by reducing the amount of DHT in the bloodstream, reduces prostatic size and MVD, ultimately reducing prostatic bleeding/haematuria [1, 15]. The duration of finasteride use varied from 2 weeks to 3 months duration [1, 15]. In fact, in an RCT comparing the impact of finasteride on prostatic bleeding, Foley et al. found that haematuria completely resolved in about 86% of patients on finasteride compared to only 37% in the control group [1, 16]. They further reported that 26% of patients in the control group required subsequent surgery where none in the finasteride group required surgery [16].

Hagerty et al. was first to report finasteride use as a pretreatment for TURP procedures [8]. They reported that of patients with >30 g of prostate tissue resected, 8.3% of patients who took finasteride 2–4 months prior TURP had perioperative bleeding compared to 36.8% who did not [8]. In addition, they reported that 16% of the patients not on finasteride required blood transfusion compared to none who were on finasteride [8].

In two recent studies, one RCT and an other an observational prospective study, aimed at exploring the role dutasteride versus placebo on reducing blood loss during and after TURP procedures, found no significance in dutasteride's use to reduce blood loss [17, 18]. Hahn et al. reported that dutasteride reduced 86–89% of the serum DHT level after 2 weeks of treatment [18]. Furthermore, they state that no significant difference was found regarding postoperative complications, but the dutasteride group did have significantly quicker operative times and less prostatic chipping weight (*P* < 0.004 and *P* < 0.001, resp.). Despite this, there was no significant difference in blood loss between the groups either during or after TURP (*P* > 0.05), in addition to no difference in the MVD between the dutasteride group and the control group (*P* > 0.05) [18]. In a study by Arratia-Maqueo et al. using dutasteride and placebo, they report no difference in blood loss after TURP (*P* = 0.88), Hb decrease (*P* = 0.73), or resected prostatic chipping weight (*P* = 0.87) [17]. However, they do mention that 33% of the patients on dutasteride had significant symptom improvement and did not require TURP [17]. This might explain why no difference was found when compared to the placebo group as the patients who improved could have altered the results. However, this is speculative, and without a large numbered RCT, this theory cannot be proven.

Limitations of this review is mainly based around the inability to meta-analyse the data, which would have clarified the dispute further. In addition, we were unable to get replies from corresponding authors of the trials despite numerous efforts. None the less, this review was conducted in an impartial and was systematically and methodically carried out in keeping with Cochrane standards. 

The disparity between the studies included in this review make it difficult to make a decisive recommendation based on the results given. However, it is evident that finasteride reduces prostatic size, as well as MVD and VEGF, which might shorten the procedure time, in addition to blood loss, clot retention, and blood transfusion requirement. However, a randomised controlled blinded trial is required with subgroups analyses based on prostate size, PSA level, and surgeon's experience.

## 5. Conclusion 

Finasteride does seem to reduce intra- or postoperative blood loss leading to less morbidity. However, further studies are required to strengthen future recommendations regarding its use as a standard pre-TURP treatment. Furthermore, a comparison between other 5ARIs such as dutasteride can also benefit the discussion and final recommendation.

## Figures and Tables

**Table:** (a)

	No (Fin versus Pl)	Age	Resection weight grams	PSA	Catheters	Prostate Ca	Complications	
Özdal et al. 2005 [10]	20 versus 20	Mean ± SD: 66.9 ± 9.43 versus 66.3 ± 5.18; ND	Mean ± SD: 23.25 ± 10.4 versus 18.73 ± 6.63; ND	Mean ± SD: 2.31 ± 2.21 versus 3.08 ± 2.07; ND			Transient (<3 months) impotence: 1 versus 0 Transient decreased libido: 2 versus 0 2 patients in the placebo group required blood transfusion	Blood loos mean ± SD: 173.47 ± 86.18 versus 235.46 ± 67.03; *P* < 0.05 Bleeding per resected gram of prostate tissue: 7.6 ± 2.37 versus 13.99 ± 4.16; *P* < 0.0001 Hb decrease: 1.88 ± 0.93 versus 3.19 ± 1.18; *P* < 0.0001

Donohue et al. 2005 [1]	31 versus 33	Mean: 70 versus 71; ND	Mean ± SD: 18 ± 14 versus 19 ± 12; ND	Mean ± SD: 9.8 ± 15.2 versus 12.6 ± 7.1; ND	10 versus 8	4 versus 6	Nil	Microvessel density mean (%CI): 60 (55–65) versus 71 (64–78); *P* < 0.01 VEGF mean (%CI): 47 (43–52) versus 61 (54–67); *P* < 0.001

Donohue et al. 2002 [7]	32 versus 36	Mean (range): 69.9 (52–81) versus 70.2 (54–86); ND	Mean (range): 17.9 (3–61) versus 18.6 (3.5–54); ND	Mean (range): 5.4 (0.6–40) versus 6 (0.4–60); ND		4 versus 6	1 transfusion in the placebo group	Hb (gm/dL) decrease mean (range): preop-recovery = 1.56 (0.1–5.1) versus 1.86 (0.8–4.4); *P* = 0.3 (ND) preop-day 1 = 0.99 (0.1–3.3) versus 1.48 (1.2–4.4); *P* = 0.6 (ND) Hb loss (gm) mean (range): irrigation fluid = 43.6 (6–182) versus 69.3 (7–228); *P* = 0.011 Gm. per/resected tissue = 2.64 (0.3–6.33) versus 4.65 (1.04–28); *P* < 0.01

Sandfeldt et al. 2001 [11]	26 versus 29	Median: 69 (56–78) versus 68 (54–76); ND	Median: 20 (12–30) versus 17.5 (10–31); ND	Median: 3 (2–5.2) versus 2.3 (2–5.8); ND		3	1 transfusion in the placebo group Readmission due to bleeding: 3 versus 2.	Blood loss: median = 279 (84–555) versus 287 (71–777) MVD = 24 (15–34) versus 22 (11–35); ND Blood loss/resection weight (mL/g) median: 14.5 (6.6–26.8) versus 16.4 (7.1–29.3); ND Blood loss/resection weight (mL/g) in prostates ≥18.6 g median: 547 versus 324; *P* < 0.01

**Table:** (b)

Özdal et al. 2005 [10]	2003-2004	5 mg finasteride for 4 weeks prior to TURP or placebo Included PSA < 4		

Donohue et al. 2005 [1]	No time	5 mg finasteride for 2 weeks prior to TURP or placebo	MVD: measured by counting stained blood vessels in 10 consecutive nonoverlapping high power fields (×200) beneath the prostatic urothelium on two slides	VEGF: (proportion of staining versus absent/faint staining)

Donohue et al. 2002 [7]	No time	5 mg finasteride for 2 weeks prior to TURP or placebo	Measured HB before and after TURP (recovery room) and the following day. Blood loss during surgery: measuring HB concentration in the irrigating fluid multiplied by the total volume of irrigating fluid used.	

Sandfeldt et al. 2001 [11]	August 1998–May 1999 Seemed to include pateints with PSAs < 2	5 mg finasteride for 12 weeks prior to TURP or placebo	Blood loss was measured by Hb concentration in each collecting bucket which was measured in duplicate by a HemoCue Low HB System, consisting of a photometer and disposable microcuvettes. The mean of the two values was multiplied by the fluid volume to yield the Hb content in each bucket. The total blood loss was obtained as the sum of the Hb content in all buckets used divided by the preoperative blood Hb concentration.	MVD: counting the number of blood vessels below the suburothelial connective tissue with an average vessel density per unit grid obtained as the mean of 30 grids per patient.

Hagerty et al. 2000 [8]	May 1996–March 1998	5 mg finasteride for 8–16 weeks prior to TURP or placebo		

NA: not available; ND: no difference between the groups.

**Table 2:** Risk of bias summary: review authors' judgements about each risk of bias item for each included study.

	Random sequence generation (selection bias)	Allocation concealment (selection bias)	Blinding of participants and personnel (performance bias)	Incomplete outcome data (attrition bias)	Selective reporting (reporting bias)	Other bias
Donohue et al. 2002 [7]			+	+	+	+
Donohue et al. 2005 [1]			+	+	+	−
Özdal et al. 2005 [10]				+	+	+
Sandfeldt et al. 2001 [11]			+	+	+	+
